# Retirement of M. Ricord

**Published:** 1861-01

**Authors:** 


RETIREMENT OF M. RICORD.—On the twenty-seventh of September, M. Ricord delivered his farewell lecture at the Hôpital du Midi, to which he had been attached for nearly thirty years, in consequence of the severe regulation now in force in Paris, compelling surgeons, officially appointed, to give up their hospitals and clinical teaching at the age of sixty. M. Ricord was born in Baltimore on the tenth of December, 1800; and pursued the more dignified course in resigning his clinical chair of syphilis, previous to the completion of his full term. The vacancy occasioned by his retirement has been filled by the appointment of M. Cusco, formerly Surgeon of the Salpetrière.

